# The Nirmiti application: An innovative tool for extending CanReg5 analyses to cancer mortality and paediatric cancer

**DOI:** 10.1002/ijc.35420

**Published:** 2025-03-29

**Authors:** Pratik Sawant, Sushama Saoba, Prithviraj Kadam, Deepak Gupta, Adarsh Kumar Ponnada, Divya Khanna, Suraj Perera, Ugyen Tshomo, Morten Ervik, Leslie Mery, Freddie Bray, Atul Budukh

**Affiliations:** ^1^ Centre for Cancer Epidemiology Tata Memorial Centre, ACTREC Navi Mumbai Maharashtra India; ^2^ Homi Bhabha Cancer Hospital & Research Centre Muzaffarpur Bihar India; ^3^ Homi Bhabha Cancer Hospital & Research Centre Visakhapatnam Andhra Pradesh India; ^4^ Mahamana Pandit Madan Mohan Malviya Cancer Centre Varanasi Uttar Pradesh India; ^5^ Strategic Information Management Unit National Cancer Control Programme Colombo Sri Lanka; ^6^ Jigme Dorji Wangchuck National Referral Hospital Thimphu Bhutan; ^7^ Cancer Surveillance Branch International Agency for Research on Cancer Lyon France; ^8^ Homi Bhabha National Institute Training School Complex Mumbai India

**Keywords:** cancer registries, incidence, LMICs, mortality, Paediatric cancer, software

## Abstract

The International Agency for Research on Cancer (IARC) Regional Hub in Mumbai provides technical support to population‐based cancer registries (PBCRs) in South and South‐East Asian (SSEA) countries. For data management and incidence rate table generation, the Hub recommends CanReg5, an open‐source registry software developed by IARC, to all PBCRs seeking support from it. However, CanReg5 is limited in generating mortality and paediatric cancer incidence tables. Several SSEA cancer registries requested the Hub to develop practical solutions to facilitate the generation of cancer rates statistics. The IARC Regional Hub, in Mumbai, subsequently developed *Nirmiti*, an innovative web application which is capable of generating incidence, mortality, and paediatric cancer rates based on provided input data. The application accepts registry data in a specific format and generates required tables according to the selected options; users can input data from CanReg5 or other software into *Nirmiti* for processing. *Nirmiti* generates childhood cancer rates for age‐groups 0–14 and 0–19, based on the 12 main groups and 47 subgroups of the International Incidence of Childhood Cancer, Volume 3, and is freely available to cancer registries upon request. The application has been successfully utilized by PBCRs in India, Sri Lanka, and Bhutan.

## Graphical abstract

What's new?

CanReg5, developed by the International Agency for Research on Cancer (IARC), is an important open‐access tool for the collection, quality assurance, and analysis of population‐based cancer registry (PBCR) data. The tool has limitations, however. The present report describes a new online application, Nirmiti, built by the IARC Regional Hub in Mumbai as a user‐friendly platform designed for improved dissemination of pediatric and mortality data. Nirmiti was effective particularly in eliminating manual errors and supporting the inclusion of childhood cancer incidence and mortality data. PBCRs in Bhutan, India, and Sri Lanka are now using the application for data management and reporting.

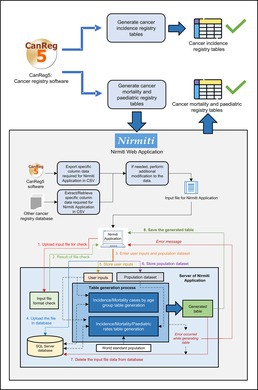

**Abbreviations**
ChildGICR: Childhood cancer Global Initiative for Cancer Registry Development
CI5: Cancer Incidence in Five Continents
CSV: Comma‐Separated Values
GICR: Global Initiative for Cancer Registry Development
IARC: International Agency for Research on Cancer
ICCC‐3: International Classification of Childhood Cancer version 3
ICD‐O‐3: International Classification of Diseases for Oncology 3rd Edition
ICD‐10: International Classification of Disease Tenth Revision
IDE: Integrated Development Environment
IICC‐3: International Incidence of Childhood Cancer, Volume 3
LMICs: Low‐ and Middle‐Income Countries
MI: Mortality‐to‐Incidence
PBCR: Population‐Based Cancer Registry
PDF: Portable Document Format
SQL: Structured Query Language
SSEA: South and South‐East Asia
WHO: World Health Organisation

## 1 INTRODUCTION

Since its inception, a primary objective of the International Agency for Research on Cancer (IARC) has been to enhance population‐based cancer registries (PBCRs) in low‐ and middle‐income countries (LMICs). Cancer data has garnered increased attention for its pivotal role in cancer control strategies and broader health system planning, resulting in a heightened demand for technical assistance. In response to the overwhelming need for robust cancer data at the population level, the Global Initiative for Cancer Registry Development (GICR) was established in 2012 to significantly improve the coverage, quality, and utilization of data from PBCRs in LMICs.^1^

The operation of PBCRs in LMICs presents specific challenges, including inadequate medical records and death registration systems, a shortage of trained registry staff, difficulties in data entry and management, and a reluctance from some key sources to provide data.^2^ In many LMICs, concerned Ministries often lack the capacity to provide necessary technical support and resources to PBCRs. To address these challenges in South and South‐East Asia (SSEA), IARC collaborated with the Tata Memorial Centre to establish an IARC Regional Hub in Mumbai, India in 2012.^3^ This Hub is tasked with providing technical support for cancer registration to countries within the region and serves as the primary point of contact for countries seeking assistance.^1^ The Mumbai Hub provides support to PBCRs across the Central SSEA region, including the countries of Afghanistan, Bhutan, Cambodia, Myanmar, India, Indonesia, Nepal, Sri Lanka, Timor Leste, and Vietnam.^4^

The Hub also facilitates the utilization of CanReg5 software, a free and open‐source tool widely adopted by PBCRs for data management, quality assurance, analysis, and dissemination. Additionally, the Hub offers technical assistance, solutions in data analysis, and report writing to PBCRs as needed.^5^ The CanReg5 software incorporates features to validate data entry, thereby ensuring the quality of the data.^6^

For the objectives of cancer control and public health, it is imperative to measure the cancer burden within a community. Accurate assessments of the cancer burden can offer a complete picture of the ways in which the effects of cancer differ between populations and geographical locations. The development and implementation of cancer control strategies are hence influenced by such data. The effectiveness of cancer control in reducing cancer burden over time often is evaluated using cancer survival. Population‐based studies commonly involve the investigation of the underlying causes, the impact of primary prevention (based on incidence), and the impact of secondary and tertiary prevention including cancer treatment and management (using survival and mortality).^7^ Using the “Table builder” feature of the CanReg5 software, a variety of standard registry tables and graphs can be created, including incidence tables of cases and rates by cancer and age for a given year.

While CanReg5 is critical for registry operations in many LMICs, it does not, at present, generate mortality tables or detailed paediatric incidence rates. As an example, the current PowerPoint presentation option within the “Table Builder” feature of the CanReg5 software generates a single slide for paediatric cancer cases aged 0–14 years. The table does not include the adolescent age range (15–19), nor does it calculate report cases or rates according to the subcategories of the International Classification of Childhood Cancer third edition (ICCC‐3). ICCC‐3 organizes tumours classified by the third edition of the International Classification of Diseases for Oncology 3rd Edition (ICD‐O‐3) into 12 primary groups, which are further subdivided into 47 subgroups. These two tiers of the ICCC‐3 facilitate standardized comparisons of childhood neoplasms, maintaining continuity with prior classifications. It adheres to existing international standards and is tailored for international, population‐based epidemiological studies, as well as cancer registries. In paediatric oncology, where case frequency is commonly low, employing an international classification system is crucial to ensure rigorous data comparability.^8^

Precise cancer mortality data is vital for assessing both cancer survival proportions and mortality: incidence (M:I) ratios. Cancer survival statistics obtained from registry data provide an estimate of the typical prognosis at the population level, potentially serving as an objective indicator of the effectiveness of cancer care among residents in the defined area.^9^ Validity and completeness are evaluated in Cancer Incidence in Five Continents (CI5) using a several semi‐quantitative indicators, including M:I ratios.^10^ Consequently, it is imperative for cancer registries to vigilantly track mortality cases from independent sources and verify the accuracy of the data.

In SSEA, several PBCRs seeking technical support from the IARC Regional Hub in Mumbai raised concerns about such limitations. The Hub's solution was an application that could generate cancer mortality and paediatric tables by ICCC‐3 entitled *Nirmiti*, a term coined from the Marathi language, as spoken in the Maharashtra state of India, conveying a meaning of “Production, creation, formation, any artistic production.” Cancer Registries from India, Bhutan and Sri Lanka are using the *Nirmiti* application for their registry data management and reporting purposes.^3^

## 2 MATERIALS AND METHODS

Cancer registry data in the region is commonly stored either within CanReg5 or maintained manually, for example, in Microsoft Excel or Access. It was crucial that mortality and paediatric tables could be generated from either source. Therefore, *Nirmiti* was developed as a website capable of accepting cancer registry data as input. The input data can be exported to CanReg5 or a manually maintained database, providing it is in the required format.

*Nirmiti* generates incidence and mortality tables including the calculation of age‐specific, crude, and age‐standardized rates as well as person‐years at risk and cumulative risk.^11^

When generating tables of paediatric cancer incidence, the classification is primarily determined by tumour morphology and primary site, with a focus on morphology rather than prioritizing the primary site as is typically done for adults.^12^ The data on paediatric cancer cases is presented using the ICCC‐3 format. The ICCC‐3 categorizes tumours based on the ICD‐O‐3 coding into 12 primary groups, further divided into 47 subgroups. These two levels of classification within the ICCC‐3 enable standardized comparisons of broad categories of childhood neoplasms, maintaining continuity with previous classifications.^8^ The data required for *Nirmiti* in the input file is demonstrated in Table 1.

**TABLE 1:** Data required and tables offered by *Nirmiti* Application.

Incidence and mortality tables (all age groups)	Paediatric tables
Data required	
Age	Age
Sex	Sex
Incidence date	Incidence date
Histology	Basis of diagnosis
ICD‐10	Behaviour
Date of death	ICCC code
Age at death	Date of death
	Age at death
Type of tables generated	
Incidence—cases by age group	Number of cases in age‐group, incidence rate per million population (Both sex)
Incidence per 100,000 by age group	Number of cases in age‐group, incidence rate per million population (By sex)
Mortality—cases by age group	Number of cases in age‐group, incidence rate per million population (Both sex) with separate zero age group
Mortality per 100,000 by age group	Number of cases in age‐group, incidence rate per million population (By sex) with separate zero age group

The input data file required for *Nirmiti* should be in a specific format because the Hub has observed that the data can be maintained in different formats in different registries. Hence the *Nirmiti* mandates its specific format in order to standardize the data. Following are the guidelines to meet the input file format of *Nirmiti*:

- The input data file is accepted in comma‐separated values (CSV) format.
- The incidence date format should be of the year(YYYY)month(MM)day(DD) format, i.e., YYYYMMDD, e.g., 19,940,930.
- The date of death format should be the same as above, but if the patient is alive or the date of death is not known, then the date should be coded as a default unknown value, 19,000,101.
- If the age at death is unknown or if the patient is alive, then the age at death should be coded as a default unknown value, 99.
- No blank data should exist for any of the column data, or else the input data file will be treated as syntactically incorrect data.

*Nirmiti* was based on a two‐tier architecture model that separates the application into two layers: the web application is the ‘client tier’, and the database is the ‘server tier’. *Nirmiti* was designed as a website, using the Dot Net framework and Microsoft SQL Server as its database. The Integrated Development Environment (IDE) used was Visual Studio 2022 Community Edition free version for Dot Net framework website front‐end and middle layer development, and licensed Microsoft SQL Server Management Studio 2017 for the database.

Upon accessing the *Nirmiti*, users are required to upload an input file for syntax verification. The *Nirmiti* then checks whether the selected input file conforms to the required format. Guidelines for the input file are provided above. If the input file adheres to the guidelines, the application displays a success message indicating the successful verification. However, if the file format is incorrect, an unsuccessful verification message is displayed, prompting the user to make the necessary modifications to the input file in accordance with the provided guidelines.

Once the input file is confirmed to be in the correct format, the user proceeds to select appropriate column names from the input files for each required field necessary to generate the table. The user also chooses the desired table type. Population datasets are then inputted into the population dataset text area, with the population dataset being in CSV format. Next, the user selects the “From” and “To” dates to specify the period for generating the table. If the selected table type is incidence, *Nirmiti* applies a filter based on the incidence date of the chosen input file. Similarly, if the selected table type is mortality, *Nirmiti* applies a filter based on the date of death from the selected input file. The user must also specify the gender if an incidence or mortality table for all age groups is selected. Finally, the user selects the export table type, either PDF or CSV, and clicks on the “Generate table” button to proceed.

During the table generation process, the *Nirmiti* initially stores the input file data temporarily in the SQL Server database. This allows for quick processing of the data. The user's selection input and population dataset are also temporarily saved to generate the table according to the user's requirements. For rate calculation, both the user‐entered population dataset and the world standard population are utilized.^13^ However, for generating tables for cases by age group for both incidence and mortality, these population datasets are not necessary. If the table requires data for a period spanning more than 1 year, such as from 2018 to 2019, then the population entered in *Nirmiti* should be the total population for both 2018 and 2019 combined. Once the table is generated, it is exported in the required file format, and the user can then download it to their computer.

To ensure data confidentiality and maintain the integrity of sensitive information, the input file data stored in the SQL Server database is automatically deleted following the successful generation of a table. This process prevents any unauthorized access and aligns with stringent data protection protocols. In the event of an exception or error during the table generation process, an error message is displayed to the user, and the input file data is promptly deleted from the database. This approach not only safeguards patient data but also ensures that no incomplete or erroneous data remains in the system, enhancing the overall security and reliability of the platform. The software developer has no right to the uploaded data, and the user has complete ownership of the data.

**FIGURE 1:**
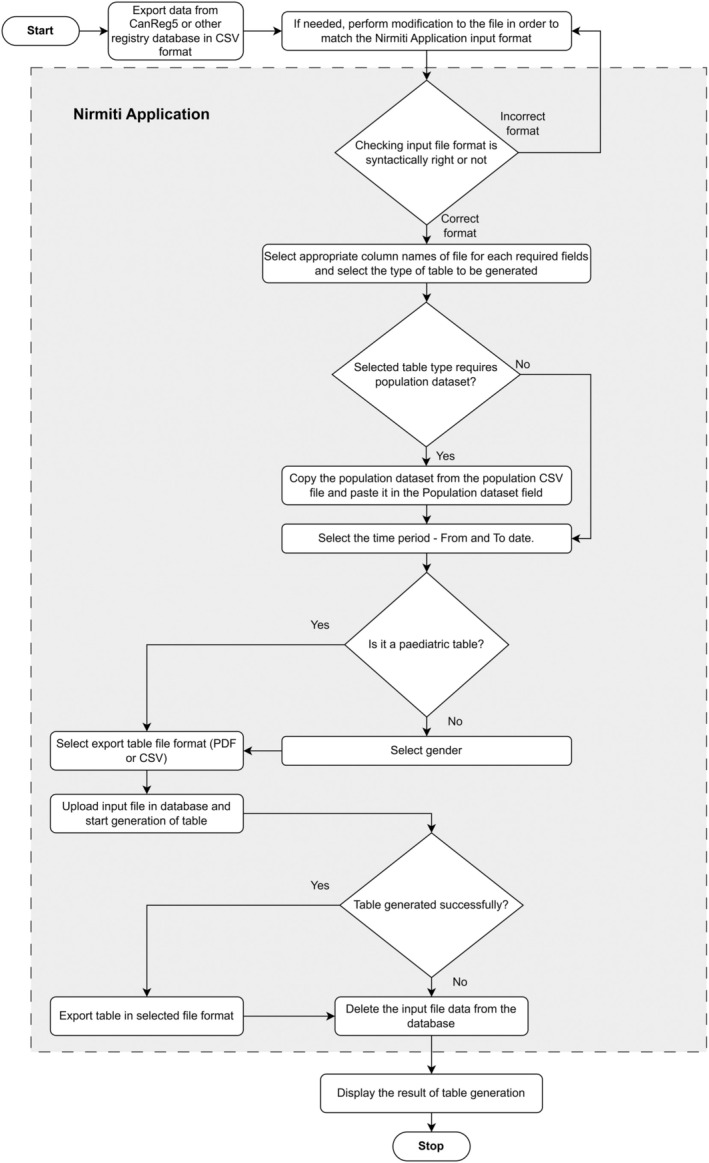
Flowchart of *Nirmiti* Application.

The entire process of table generation through the *Nirmiti* is illustrated in Figure 1, Flowchart of the *Nirmiti* Application. For the incidence and mortality, the tables offered by the *Nirmiti* are mentioned in Table 1.

Initially, the website was tested by the technical team at the IARC Regional Hub, Mumbai, and minor errors were observed and modified accordingly.

**FIGURE 2:**
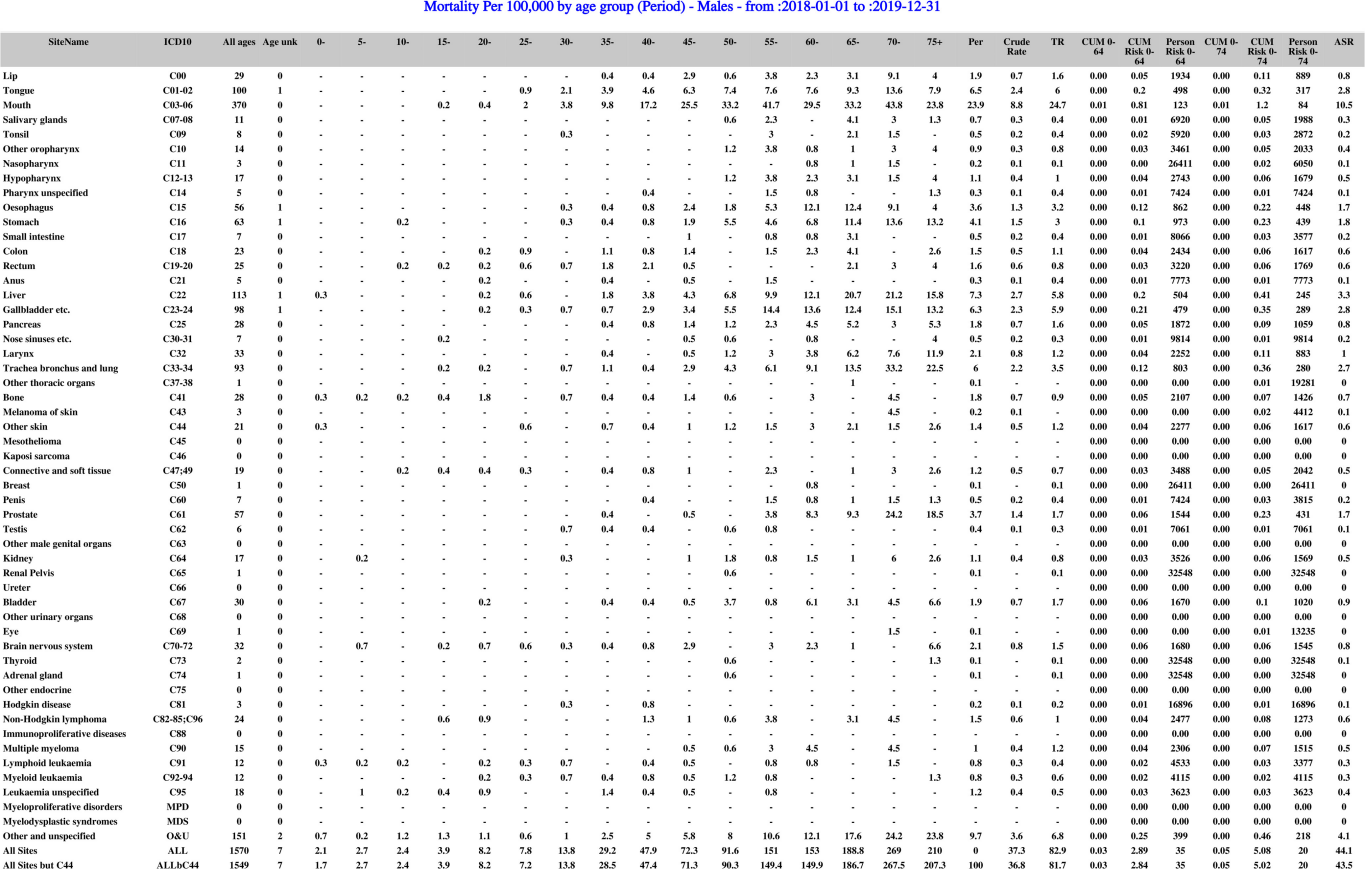
Mortality per 100,000 by age group—Males, year 2018 to 2019, Varanasi PBCR, India.

**FIGURE 3:**
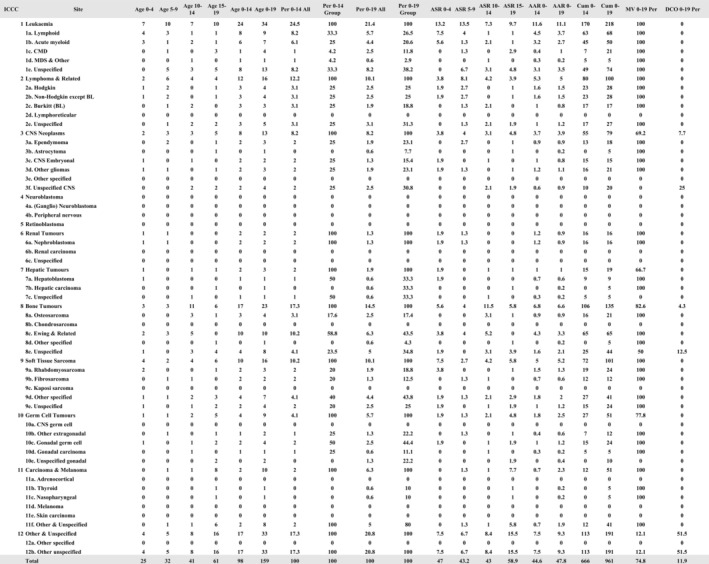
Paediatric Number of cases in age group (both sexes), 2018–2019, Varanasi PBCR, India.

Figures 2 and 3 represent the outputs of *Nirmiti*, derived from registry data of Varanasi PBCR for the years 2018 and 2019. Figure 2 displays mortality data within the Portable Document Format (PDF) exported from *Nirmiti*, while Figure 3 presents the paediatric data rates table exported in CSV format. Figure 3 is formatted in Microsoft Excel according to user preferences and requirements.

In Figure 2, the left column lists all cancer sites with their ICD‐10 codes, along with respective data, including age‐specific rates from ages 0 to 75+ in 5‐year intervals, crude rate, truncated rate (TR), cumulative rate and risk for ages 0 to 64 and 0 to 74, person risk, and age‐standardized rate (ASR). In Figure 3, the left side displays the 12 main groups and 47 subgroups from the International Incidence of Childhood Cancer, Volume 3 (IICC‐3), alongside the number of cases for ages 0 to 19 in 5‐year intervals and for age ranges 0 to 14 and 0 to 19. Additional metrics include the percentage of cases by age group, age‐specific rates, age‐adjusted rates (AAR), percentage of microscopic verification (MV), and death certificate only (DCO) percentage.

## 3 RESULTS

The *Nirmiti* was introduced to SSEA Cancer Registries during the “Data Management of PBCR” workshop in September 2023, hosted by the IARC Regional Hub Mumbai. Several Indian PBCRs, including Vizag, Varanasi and Muzaffarpur, successfully utilized the *Nirmiti* subsequently generating various tables during the preparation of their interim reports. Figures 2 and 3 are the registry standard mortality and paediatric rates tables generated from *Nirmiti* for Varanasi PBCR, India. Additionally, PBCRs from the SSEA region, including Sri Lanka and Bhutan, incorporated *Nirmiti* into their data monitoring and management processes. These registries also integrated the tables generated by the *Nirmiti* into their interim reports.

Creating a cancer incidence or mortality rate table by gender for a specific year involves calculating age‐specific rates, crude rates, age‐standardized rates, truncated rates, cumulative rates, and risks for 53 cancer sites across age groups from 0 to 75+. Similarly, producing a paediatric cancer rate table involves calculating age‐specific rates, age‐adjusted rates, cumulative risk, MV, and DCO cases for ages 0–14 and 0–19, covering the 12 main groups and 47 subgroups in the IICC‐3. For all these rate calculations, population data by age group for the PBCR region for the specified year is required as a denominator, in addition to the world standard population for standardization of rates.

Registries have reported that it typically takes more than 3 h to manually generate incidence, mortality, or paediatric tables. The time taken to generate tables was monitored to assess the efficiency of *Nirmiti*. Registry personnel with minimal knowledge of cancer statistics used *Nirmiti*, and it took an average of 15 min to generate a table, including data exporting from CanReg5 and making minor modifications to the exported data. Manual generation using spreadsheets for more than 3 h of person time leads to significant inefficiencies. The process is prone to manual errors when calculating rates, resulting in high data inconsistency. Additionally, the complexity and time‐consuming nature of the manual process cause delays in data analysis.

**FIGURE 4:**
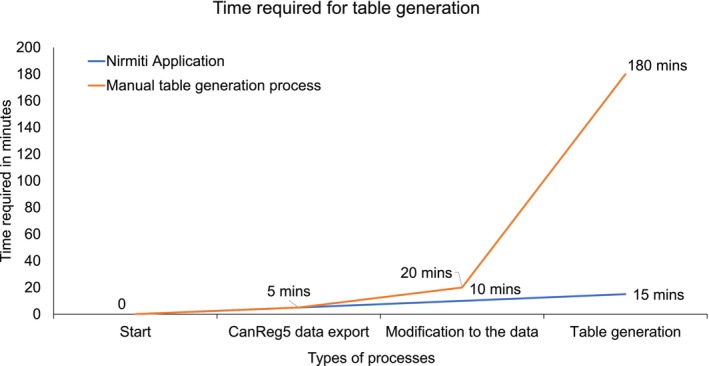
Comparison of time required for table generation between manual process and *Nirmiti* Application.

Figure 4 illustrates a comparison of the time required for table generation between the two processes. With automatic generation using the *Nirmiti*, the process takes approximately 15 min and helps to eliminate errors and data inconsistencies.

## 4 DISCUSSION

In 2025, more than 20 million new cancer cases are expected worldwide, with about four‐fifths occurring in LMICs. To tackle the increasing cancer rates effectively, planners must obtain accurate and unbiased data on the cancer burden in their communities.^14^ Planning cancer control initiatives without dependable data from cancer registries can lead to misdirected focus and squandered resources.^14^ In LMICs, the burden of cancer cases is high, but the coverage of the same in CI5 is less. To address current gaps in childhood cancer registration, IARC and St. Jude Children's Research Hospital recently launched the Child Global Initiative for Cancer Registry Development (ChildGICR). This program aims to boost the sustainable development of PBCRs in LMICs and establish evidence‐based standards for national childhood cancer registration.^15^

In CanReg5, there is no provision at present to generate the mortality tables or provide the paediatric rates as per the ICCC3 subcategories for 0–19. The registry staff has to handle the data analysis task manually, which is a time‐consuming process prone to errors. Registry personnel from LMICs have technical limitations in data analysis and face difficulties in creating the cancer mortality (all sites) and paediatric incidence rates tables. Such a table creation process is complex and subject to human error and inaccuracies. *Nirmiti* thus serves as a valuable tool for registries in LMICs in filling the present gap; the tables seek to inform policymakers and paediatric oncologists alike.

In response to user requirements, new tables can be added to *Nirmiti*, enhancing its flexibility and adaptability for various data needs. Additionally, the application's capability to generate childhood cancer incidence and mortality rates can be integrated as an added feature of CanReg5. By incorporating these advanced features into CanReg5, cancer registries can benefit from more comprehensive data analysis, including critical childhood cancer and mortality metrics, further enhancing the efficiency of cancer data management and reporting.

*Nirmiti* has some limitations. Users must make additional data modifications to be able to work with *Nirmiti*. Currently, the application is only accessible online; as users have expressed a need for an offline version of the software, this is being investigated.

As an integral part of the GICR, the IARC Regional Hub in Mumbai plays a significant role in capacity building and offering technical solutions to PBCRs. These services are provided free of charge. The cancer registry teams of Afghanistan, Bhutan, Cambodia, Myanmar, Nepal, Sri Lanka, Timor‐Leste, and Vietnam received in‐person training. To date, the Hub has conducted 47 courses, both in‐person and virtually, and has trained 1200 participants across the SSEA region.^4^ Continuous technical support and training for cancer registration and data management are provided by the Hub whenever requested by the respective registry. SSEA cancer registries from Nepal, Afghanistan, Myanmar, Timor Leste, Bhutan, Indonesia, Thailand, Korea DPR, Vietnam, Cambodia, and Maldives are encouraged to reach out to the IARC Regional Hub in Mumbai for assistance as needed.

To conclude, the *Nirmiti* application has demonstrated its value in analyzing cancer registry data, in eliminating manual errors, and supporting the inclusion of childhood cancer incidence using IICC‐3 as well as mortality data. This ensures that cancer registries operate efficiently, facilitating progress in data management, analysis, and reporting. *Nirmiti* is freely available to all cancer registries, and those within the SSEA region can contact the IARC Regional Hub in Mumbai (budukham@tmc.gov.in) for technical support with *Nirmiti*, as well as CanReg5 software, cancer registry training, data analysis, and collaboration in developing registry reports and scientific publications utilizing the collected data.

## AUTHOR CONTRIBUTIONS

**Pratik Sawant:** Visualization; methodology; validation; software; formal analysis; writing – original draft; writing – review and editing. **Sushama Saoba:** Methodology; writing – review and editing; validation; formal analysis. **Prithviraj Kadam:** Validation; formal analysis. **Deepak Gupta:** Validation; formal analysis. **Adarsh Kumar Ponnada:** Validation; formal analysis. **Divya Khanna:** Validation; formal analysis; writing – review and editing. **Suraj Perera:** Validation; formal analysis; writing – review and editing. **Ugyen Tshomo:** Validation; formal analysis; writing – review and editing. **Morten Ervik:** Validation; formal analysis; writing – review and editing. **Leslie Mery:** Validation; formal analysis; writing – review and editing. **Freddie Bray:** Validation; formal analysis; writing – review and editing. **Atul Budukh:** Supervision; conceptualization; project administration; writing – review and editing.

## CONFLICT OF INTEREST STATEMENT

The authors declare that they have no conflict of interest.

## DISCLAIMER

Where members are identified as personnel of the International Agency for Research on Cancer/World Health Organization, the authors alone are responsible for the views expressed in this article and they do not necessarily represent the decisions, policy, or views of the International Agency for Research on Cancer/World Health Organization.

## Data Availability

Users can access the *Nirmiti* via the link https://medrecs.actrec.gov.in/nirmiti. Access to the application is restricted to registered users, who authenticate themselves through a login ID and password. *Nirmiti's* login page is represented in Figure 5. Further information is available from the corresponding author upon request. Login page of *Nirmiti* Application.
